# Secondary Dentine

**Published:** 1882-05

**Authors:** John G. Harper


### ARTICLE III.

### *Secondary Dentine.*

BY JOHN G. HARPER, D. D. S.

[ Read before the St. Louis Dental Association, February 21, 1881. ]

The pathological changes to which the dental pulp is subjected are many, and of the greatest importance to patient and practitioner. They vary from the reparative to the destructive. The particular pathological change to be considered here is the calcification of this delicate organ. A normal pulp which has never been subjected to sufficient irritation to cause any changes in its form or structure, presents a mass which is soft, vascular and nervous, possessing a function which may, in the future, form dentine similar to that of the tooth, and called secondary dentine.

Salter, page 140, says; "The pathological change consists in the impregnation of the various tissues of the tooth-pulp with calcareous matter, their calcification, in fact, occurring in multitudes of isolated points; and by the multiplication and enlargement of these 'islands of calcification,' involving more and more of the structure of the pulp, and its ultimate conversion, under certain favorable circumstances, into osteo-dentine." * * * "When the calcification is absolutely complete, * * the whole is perfectly hard as ivory, but, until this final stage, it may be torn up with points of needles, into fibers, which in the fangs are uniformly longitudinal. The axis of the pulp solidifies first, and most completely, the exterior being more or less soft and pulpy, till the osteo-dentine is fused and confounded with the dentine."

* * * "The color is also modified by the change, according to the degree of calcification that has taken place; from pink and semi-transparent, it becomes white and opaque, and when the calcification is complete, and the islands have fused together, it is yellowish and horny-looking." * * * "The blood vesssls are probably the last tissues to calcify, and this is certainly the case with the larger ones, some of which remain permanently patent and functional in the axis of Haversian systems of the matured osteo-dentine."

Secondary Dentine may be divided into three classes, viz.: Dentine of Repair, Dentine Excrescence, and Osteo-Dentine, or Intrinsic Calcification.—(Tomes.)

Dentine of Repair is formed for the protection of the dental pulp from external influences, and we know of no case where any disturbance has been created by its formation.

Dental Excrescences are little nodules of secondary dentine formed upon the walls of the pulp chamber of the teeth which have been subject to no other apparent change or lesion.

They seldom give any pain, but sometimes are the cause of neuralgia. As the first form of secondary dentine never gives uneasiness, and the second seldom, we will pass them by and take up more minutely the third class, or Osteo-Dentine, which very often required the attention of the practitioner of dentistry.

"All the tissues appear to be calcified promiscuously; the vessels, or many of them, are the last affected, still they are early reduced in number, as those which occupy the axis of the dentine—Haversian systems—are far less numerous than those of the original pulp." (Salter, p. 69.) * * * "Osteo-dentine has fewer tubes than any other form of dentine, and is usually very transparent." (Salter, p. 70.)

The tubes in osteo-dentine are irregular in direction, which is easily accounted for by referring to the vessels and nerves in a normal pulp.

Calcification of the dental pulp is confined to no particular period of life. It is found in deciduous teeth, and in the permanent teeth of the young, the middle aged, and the aged. From observation, I should conclude that the deciduous teeth are oftener found in this condition than the permanent of any age.

As this condition of the pulp is a pathological one, we will look for the causes.

Irritation no doubt is the cause, and this may be produced in a number of ways, viz.: Caries, which removes a part of the tooth, and exposes the dentine to outside influences which act directly upon the pulp; recession of the gums and alveolar process; abrasion, chemical or mechanical; large metallic fillings and lack of occluding teeth. Tomes, p. 475, says: "In old age the pulp, to some extent, shrivels, and becomes the seat of various degenerative processes. Thus the arteries and veins become indistinguishable, and their coats are kept rigid and distended by irregular calcareous depositions upon them." * * * "These same results may follow upon irritation of the pulp, even in young people."

What are the symptoms by which we may be enabled to diagnose this pathological condition of the dental pulp? The answer is obtained by careful study and close observation of cases. But first let us consult good authority. Tomes (p. 575,) in giving the causes of neuralgia, mentions secondary dentine in the pulp-cavity. Neuralgia is, therefore, one symptom: and he says, quoting Dr. Cayley, "It may be here mentioned that the attack, when it is dependent on diseased teeth, almost always comes on in the evening." He also says, "As a general rule, the pain due to osteo-dentine is of gradual development." * * "It is usual for pain due to partial calcification to be distinctly localized, so that the patient is enabled to point out the affected tooth." Sharp, lancinating pains, and sensitiveness to thermal changes, are other symptoms.

Neuralgia is one of the symptoms that has been accepted as belonging to calcification of the dental pulp, and, no doubt, the pain is caused by the nerves becoming irritated by the calcific deposits, or the entangling of the nerves among the nodules of osteo-dentine. In Tomes (p. 571) we find the following, which I will present as collateral evidence of this theory: "A remarkable form of neuralgia has recently been described by Dr. Gross, Professor of Surgery, as occuring in edentulous jaws, or in spaces from whence teeth have been removed. * * * The explanation of the pathology of the affection, offered by Professor Gross, is that the minute nerves, distributed through the wasted alveolar border, have undergone compression from the osseous matter in the canals, and some support is lent to this view by the fact that the bone was found to have a dense ivory like consistence, where cut down upon at the affected spots, and the overlying gum was dense and unusually adherent."

"In each case recorded, Professor Gross, after the failure of other remedies, resorted to the excision of the affected portion of the alveolus, which, in most cases, affected a permanent cure, and in all produced great alleviation of the symptoms." Also we may add an itching sensation, simulating that felt in the knitting of a broken bone. I will offer the following theory to account for the sharp sudden, lancinating pain felt in a calcified pulp. The symtom will be observed in a case where there has been a dull pain for some time, due to an inflamed condition of the remaining soft part of the pulp; the small blood vessel, the artery or capillary, has become so small, or hardened, that it is with difficulty the blood corpuscle, *or a clot*, can pass through, and it becomes lodged; but finally the *mass suddenly passes through*, and being forced upon the inflamed mass, causes this sharp pain. If the case is allowed to run its course, inflammation of the remaining portion of the pulp results in its death, and the decomposition will cause inflammation of the periosteum, which may result in an abcess, and this finally coming to the surface of the gum, gives vent to the pus, and relief to the patient.

Grouped together, the symptons are neuralgia, sensitiveness to thermal changes, "slight uneasiness at any time," lancinating pains and itching. In the last stages, pericementitis and its sequences.

The treatment, when there is no occluding tooth, and is not desirable, or practical, to insert one, as, for instance, a wisdom tooth, extract.

Should it be desirable to preserve the tooth, cut into the pulp chamber, devitalize, if alive, or remove the decomposed mass, if dead, treat in the usual manner. Insert occluding teeth, when caused by their lack.

### CASES.

Miss ———, between twenty-five and thirty years of age, ame to have some teeth filled. Found the left inferior first molar with a large amalgam filling in crown surface, which had been inserted five or six years before. The tooth had decayed on the distal surface until the pulp was almost exposed; filled with oxy-phosphate as a temporary filling, and had no further trouble. This was in August, 1880. Called again, April 11, 1881; complained of pain in teeth and face on left side, but could not locate in any particular tooth; found temporary filling in good condition. Complained of a feeling of pressure in the tooth under consideration. During the time between May, 26, 1881 and July 3, she called several times, and I had separated the tooth from the one in front and on distal surface, but still the feeling of pressure remained; and at one of the visits she said that the day before she felt a sudden, severe pain that was almost enough to make her faint. Proceeded to devitalize the pulp; but still the feeling of pressure remained. Made up my mind that it was a case of exostosis, and extracted, and found nodules of osteo-dentite in pulp, and roots largely exostosed. The patient had no further trouble.

In Salter (p. 137) is the following: "In one case, a little boy, eight years of age, was suffering extreme pain from an apparently sound temporary canine. Mr. White extracted the tooth and the pain ceased. In the pulp cavity was found a lobulated, calcified mass, filling nearly the whole cavity at the junction of its middle and lower thirds, and pressing the *fasciculi* of nerve fibres out of their course."

In 1875 I had a case of which I made a few notes at the time. The patient, a man, age twenty-eight; the tooth, a first superior molar, left side; had been filled six years before, with amalgam; filling had been out two years; tooth had ached slightly at times, since filling came out. In preparing to refill, the horn of the pulp was exposed. The tooth was medicated, and filled temporarily. In a week, during my absence, the tooth began to ache badly, After a week of suffering, the plug was removed, and the pulp found partially dead. After a few days the dead pulp was removed, and several nodules of calcified matter were found. The pulp in buccal roots was soft and vascular, and that in the palatal, calcified.

In a discussion which followed, Dr. W. H. Eames said that there was an objection to using the term Osteo-Dentine as applied to this texture by Tomes, as Osteo-Dentine—bone and dentine commingled—was seldom found in human teeth, but quite frequently in those of some animals.

Dr. Harper thought that Tomes used Osteo-Dentine and Intrinsic Calcification synonymously, as this form of Secondary Dentine possessed, in its form, certain characteristics of bone, but not necessarily bone cells.—*Ohio State Journal of Dental Science.*

---

